# Treatment resistance of rheumatoid arthritis relates to infection of periodontal pathogenic bacteria: a case–control cross-sectional study

**DOI:** 10.1038/s41598-022-16279-z

**Published:** 2022-07-19

**Authors:** Kazu Takeuchi-Hatanaka, Yoshinobu Koyama, Kentaro Okamoto, Kyosuke Sakaida, Tadashi Yamamoto, Shogo Takashiba

**Affiliations:** 1 Department of Periodontics and Endodontics, Okayama University Hospital, Okayama, Japan; 2 Center for Autoimmune Diseases, Division of Rheumatology, Japan Red Cross Okayama Hospital, Okayama, Japan; 3 Department of Pathophysiology-Periodontal Science, Okayama University Graduate School of Medicine, Dentistry and Pharmaceutical Sciences, 2-5-1 Shikata-cho, Kita-ku, Okayama, 700-8525 Japan

**Keywords:** Rheumatoid arthritis, Periodontitis, Prognostic markers, Infection

## Abstract

Recent studies have shown that periodontitis is associated with rheumatoid arthritis (RA) and periodontal bacteria, such as *Aggregatibacter actinomycetemcomitans* (*Aa*) and *Porphyromonas gingivalis* (*Pg*) are involved in the pathogenesis of RA via citrullinated proteins. Smoking has also been shown to be involved in the pathogenesis of RA; however, the extent of this involvement is still poorly understood. In addition, RA and polymyalgia rheumatica (PMR) are sometimes difficult to differentiate; however, the relationship between PMR and the factors from smoking and periodontal bacteria is unclear. The aim of this study was to clarify the relationship between periodontal pathogenic bacterial infections and smoking in patients with RA or PMR. This case–control study included 142 patients with untreated RA or PMR. This study evaluated the serum antibody titers against periodontal pathogenic bacterial antigens and an anti-citrullinated peptide antibody (ACPA). In patients with RA, the relationship between antibody titers and disease activity of RA and response after 3 months of treatment was also investigated. Additionally, the effects of smoking were evaluated. Although there was no significant difference in serum antibody titer against periodontal pathogenic bacteria between the ACPA-positive RA group and the ACPA-negative PMR group, we found an association between the elevated antibody titer against *Pg* and the degree of ACPA value, especially between negative group and high-value positive group (≥ 100 U/mL). The antibody titers against *Aa* and *Pg* did not differ depending on disease activity score 28 (DAS28) at baseline; however, patients with high antibody titers had poor RA therapeutic response as judged by DAS28 after 3 months. We could not find any association between smoking and any of these parameters. Periodontal pathogenic bacteria, especially *Pg*, are associated with elevated ACPA levels. Our findings suggest that *Pg* and *Aa* infections interfere with the therapeutic response of RA.

## Introduction

Rheumatoid arthritis (RA) and periodontitis are both chronic inflammatory diseases associated with bone destruction, release of inflammatory cytokines, and osteoclastic activity, and smoking is a common risk factor for both diseases^1–3^. Several previous studies have reported a high prevalence of mutuality between RA patients and periodontitis patients^4–8^. A meta-analysis found that patients with RA had an approximately 13% greater risk of developing periodontitis than healthy controls, ranging from 4 to 23% (relative risk: 1.13; 95% confidence interval [CI]: 1.04, 1.23; p = 0.006)^9^. Periodontitis is a disease that is initially caused by infection with periodontal pathogenic bacteria, and measurement of serum IgG antibody titers against these bacteria is one of the periodontal examinations^10,11^. For rheumatologists, who have difficulty obtaining clinical data of periodontitis themselves, the use of blood test is an easy and effective means of ascertaining the extent of periodontitis without oral examination.

*Porphyromonas gingivalis* (*Pg*), a periodontal pathogenic bacterium, is the only bacteria in the oral cavity with the enzyme that converts arginine to citrulline^12^. It has been proposed that when infected with *Pg*, a periodontal tissue protein is citrullinated by this enzyme and the resultant anti-citrullinated protein antibody (ACPA) forms an immune complex with citrullinated proteins in the joints, thereby inducing arthritis^13,14^. ACPA is a main marker of RA with a sensitivity of 70% and specificity of 90%. Previous studies reported that serum immunoglobulin G (IgG) antibody titer to *Pg* was significantly higher in patients with RA than that in those without RA^15,16^. A series of studies on the onset and progression mechanism of periodontitis and RA showed that the serum antibody reactions to arginine converting enzyme of *Pg* may reflect reactivity to anti-rheumatic drugs^15,17^.

*Aggregatibacter actinomycetemcomitans* (*Aa*), a periodontal pathogenic bacterium, has an influence on activation of citrullinating enzyme by producing an exotoxin in the form of a leukotoxin^18^. However, it was reported that the serum antibody titer to *Aa* in patients with RA were significantly lower than those in controls^15^. In addition, metagenomic sequencing and polymerase chain reaction analyses in subgingival plaque have not revealed a significant association between the presence of *Aa* and RA^19,20^.

RA has some genetic background^21^, and in particular, the association of the HLA-DRB1 allele, which contains a specific amino acid motif called the shared epitope (SE), with disease susceptibility and severity of RA has received considerable attention^22^. It was later reported that the association between RA and SE is more pronounced in the ACPA-positive group than in the ACPA-negative group^23^. In addition, there are seemingly contrasting genetic backgrounds and different underlying etiologies in patients with ACPA-positive and -negative RA^24^. The presence and levels of ACPA have been reported to be associated with periodontal conditions in patients with RA^25^. As a comparative disease of RA, we focus on polymyalgia rheumatica (PMR), a collagen disease that is often difficult to distinguish from RA. PMR is common in the elderly and is associated with inflammatory reactions, such as increased erythrocyte sedimentation rate (ESR) and elevated C-reactive protein (CRP) levels; however, rheumatoid factor (RF) and ACPA are usually absent. To date, no studies have reported the relationship between periodontitis and PMR. The originality of this study lies in the comparison of IgG antibody titers against periodontal bacteria between the patients suffering from ACPA-positive RA and those suffering from ACPA-negative PMR, analogous disease of RA as controls. RA activity is known to affect periodontal disease. Rodríguez-Lozano et al. reported a significant association between the severity of periodontitis and RA disease activity^26^. Another report showed that periodontitis affects the therapeutic response to biologics used in the treatment of RA^27^. Therefore, we investigated the disease activity and therapeutic response of RA and the antibody titer against periodontal pathogenic bacterial antigens in patients with RA. The elucidation of the relationship between periodontitis and RA through the origin, disease activity, and therapeutic response will contribute to future medical advances.

The aim of this study was to clarify the relationship between periodontal pathogenic bacterial infections and smoking in patients with RA or PMR.

## Methods

### Study population

Japanese patients with RA or PMR who first visited the Japan Red Cross Okayama Hospital between March 2012 and February 2018 without any treatment history were all enrolled in this study. Their sera were provided by the "sample bank" of the hospital. RA was diagnosed based on the results of 2010 American College of Rheumatology or the European Alliance of Associations for Rheumatology (ACR/EULAR) classification criteria^28^. PMR was diagnosed based on the results of 2012 EULAR/ACR provisional classification criteria^29^.

### Study design

This was an observational, case–control cross-sectional study of patients with RA and PMR. The titers of ACPA were measured using Architect Anti-CCP (Abbott Japan LLC, Tokyo). The standard criteria for ACPA negative and positive is 4.5 U/mL, which is the standard of most laboratories employed in Japan. The criteria for positive and high positive are based on Ref.^30^. The disease activity score 28 (DAS28-CRP and DAS28-ESR) was calculated using the DAS-score website (http://www.das-score.nl/). Based on the amount of improvement in DAS28 after 3 months, RA therapeutic response was classified according to the EULAR criteria^31,32^ as good, moderate, or no response. To simplify the result of therapeutic response, we also categorized patients into two groups based on the DAS ratio (DAS ratio = DAS28-ESR or CRP at 3 months/DAS28-ESR or CRP at baseline: good responders < 0.75 and poor responders ≥ 0.75)^33^. In addition, smoking status was questioned and classified into three categories: current, former, and never.

**Figure 1:**
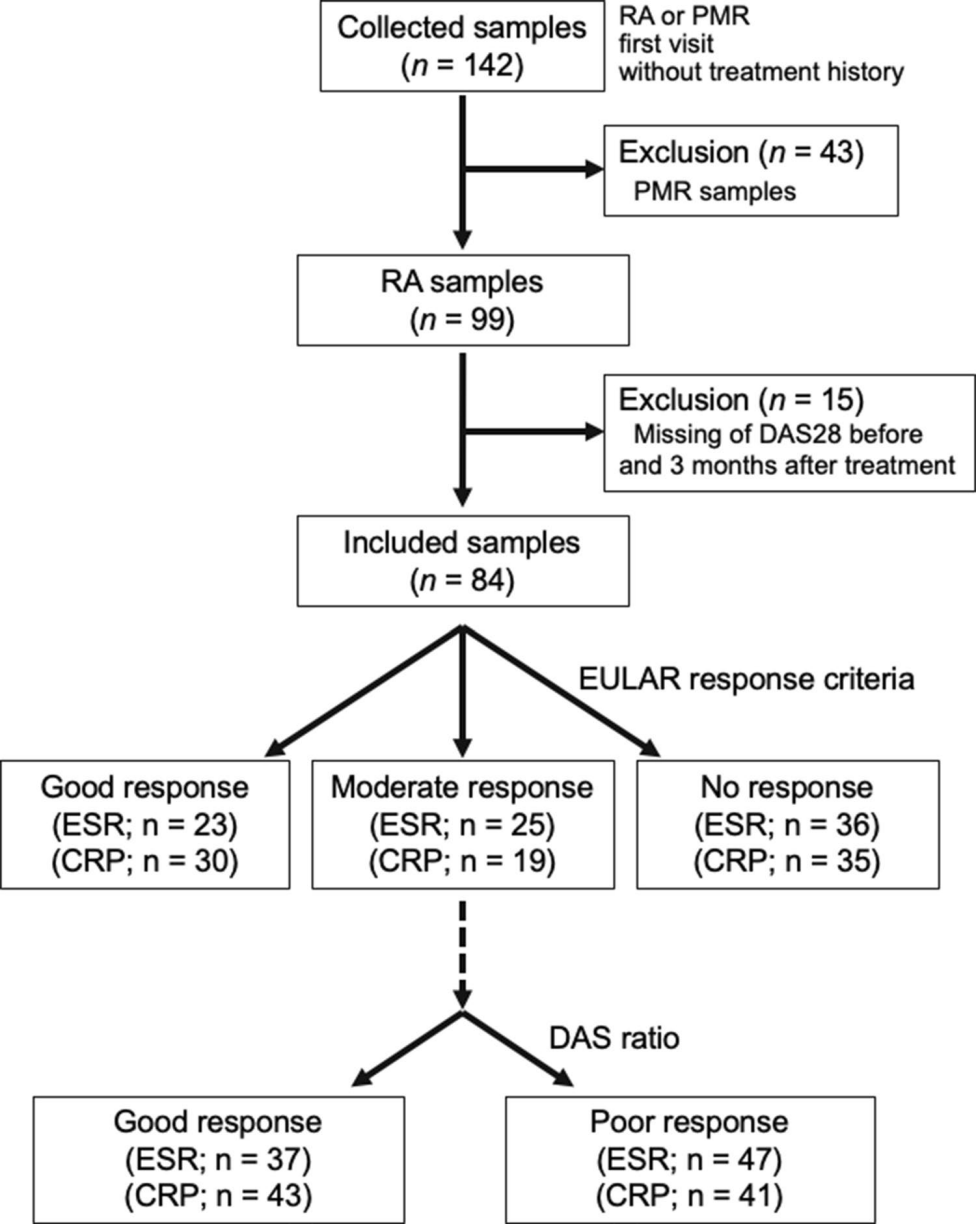
Flow chart of analyzed RA samples based on therapeutic response. Serum IgG antibody titers were classified according to the therapeutic response of RA into two groups and analyzed. After 3 months of treatment, the patients were grouped according to the EULAR response criteria (good, moderate, no response) and the DAS ratio (good and poor response) using DAS28-ESR and DAS28-CRP. *CRP* C-reactive protein, *DAS28* disease activity score 28, *ESR* erythrocyte sedimentation rate, *EULAR* European Alliance of Associations for Rheumatology, *IgG* immunoglobulin G, *RA* rheumatoid arthritis.

Patients were classified according to the disease, RA or PMR (Supplemental Fig. S1), and the degree of ACPA titers (Supplemental Fig. S2). Furthermore, patients with RA were classified according to DAS28-CRP or DAS28-ESR (Supplemental Fig. S3). In addition, they were classified according to the therapeutic response, as determined by the changes in DAS28 after 3 months (Fig. 1). The exclusion criteria were first to exclude samples with missing values for each of them. After that, the flowchart is as shown in Fig. 1 and supplemental Figs. S1–S3. The patients' serum IgG titers against periodontal pathogenic bacterial antigens were analyzed.

### Measurement of serum IgG titers against periodontal pathogenic bacterial antigens

Serum IgG titers against the crude extracts of sonicated periodontal bacteria were measured using enzyme-linked immunosorbent assay (ELISA), as previously described^34,35^. All patients' sera were obtained at just before treatment after diagnosis. Sonic extracts of periodontal bacteria, including *Aa* Y4, *Aa* ATCC29523, *Aa* SUNY67, *Pg* FDC381, and *Pg* SU63, were used as antigens for ELISA. In this study, the average antibody titers against these three strains of *Aa* and two strains of *Pg* were used. Antibody titer was defined using the following formula: titer = (ELISA unit of the patient – mean of the healthy controls) / 2 standard deviations [SD] of the healthy controls. The healthy controls were 10 generally healthy adults without periodontitis, and their pooled-sera were used for drawing calibration curve^35,36^.

### Statistical analysis

To understand whether age and smoking affect RA parameters and the degree of infection with periodontal bacteria, their correlation coefficients were examined. The serum IgG antibody titers of each group were compared using Student’s *t*-test or one-way analysis of variance and post-hoc analysis (Bonferroni test). In each comparison, smoking status was examined using Fisher's exact test or Pearson’s chi-square test. The relationship between therapeutic response and periodontal bacterial infection and smoking was analyzed using Pearson's chi-square test, as shown in the Marimekko Chart. In order to evaluate the relationship between therapeutic response of RA (objective variable) and periodontal bacterial infection (explanatory variables; IgG titers against Pg and Aa) with adjusted factors (age, sex, ACPA, and smoking), a multivariate analysis was performed using logistic regression models. In these analyses, statistical significance was set at P < 0.05.

The statistical software JMP version 9.0.2 (SAS Institute Inc., Cary, NC, USA) and NCSS version 2021 (JUCA, Inc., CA) were used to analyze the data.

### Ethics approval and consent to participate

We confirm that all methods employed in this study were carried out in accordance with relevant guidelines and regulations (Declaration of Helsinki). This study protocol was approved by the Ethics Committee of Okayama University Graduate School of Medicine, Dentistry and Pharmaceutical Sciences (acceptance number 1709-040). Written informed consent was obtained from all study participants for using their samples from the "sample bank" of the Japan Red Cross Okayama Hospital.

### Consent for publication

In this manuscript, individual patient data are not presented.

## Results

### Target sample of the study

One hundred forty-two samples (from 47 men and 95 women, average age 64.9 ± 15.1 years) were collected for this study. In the characteristics of all patients, median ACPA, ESR, CRP, and RF values were far above the normal values (Table 1). First, serum IgG antibody titers against periodontal bacteria in patients with PMR and RA were measured (Supplemental Fig. S1). Thirty-eight samples of the PMR group were ACPA negative and 82 samples of the RA group were ACPA positive. Next, antibody titers were analyzed based on the degree of ACPA titers (Supplemental Fig. S2). The ACPA level was < 4.5 U/mL in 53 samples, ≥ 4.5 U/mL or < 100 U/mL in 29 samples, and ≥ 100 U/mL in 58 samples. Furthermore, antibody titers of patients with RA were analyzed based on disease activity (Supplemental Fig. S3). The DAS28-CRP level was ≥ 2.3 in 81 samples and was compared between three groups, 2.3–2.7; 2.7–4.1; and ≥ 4.1. The DAS28-ESR value was ≥ 2.6 in 86 samples and was compared between three groups, 2.6–3.2; 3.2–5.1; and ≥ 5.1. Therapeutic responses in patients with RA grouped by EULAR response criteria were good (23 by DAS28-ESR and 30 by DAS28-CRP), moderate (25 by DAS28-ESR and 19 by DAS28-CRP), and no response (36 by DAS28-ESR and 35 by DAS28-CRP). They were also classified according to the DAS ratio (= DAS at 3 M /DAS at baseline) as good (< 0.75: 37 by DAS28-ESR and 43 by DAS28-CRP) and poor response (≥ 0.75: 47 by DAS28-ESR and 41 by DAS28-CRP) (Fig. 1).

**Table 1:** Patient characteristics and clinical parameters.

Factor	Value
Sex	Female 95 (67%), Male 47 (33%)
Mean Age (SD)	64.9 years (15.1)
Smoking	Current 16 (11%), Former 36 (25%), Never 90 (63%)
Median ACPA (*P*_*25*_; *P*_*75*_; Ref.)	91.7 U/mL (2.2; 401; 4.5)
Median ESR (*P*_*25*_; *P*_*75*_; Ref.)	51 mm/h (27; 80; 20)
Median CRP (*P*_*25*_; *P*_*75*_; Ref.)	1.48 mg/dL (0.46; 4.56; 0.3)
Median RF titers (*P*_*25*_; *P*_*75*_; Ref.)	80 IU/mL (28; 176; 15)

Data represent numbers (percentages), mean (SD), or median (*P*_*25*_; *P*_*75*_).

*ACPA* anti-citrullinated peptide antibody, *CRP* C-reactive protein, *ESR* erythrocyte sedimentation rate, *RF* rheumatoid factor, *SD* standard deviation.

*P*_*25*_ 25th percentile, *P*_*75*_ 75th percentile, *Ref.* reference value.

### Possible influence of factors, such as age and smoking, on the pathophysiology of RA

**Figure 2:**
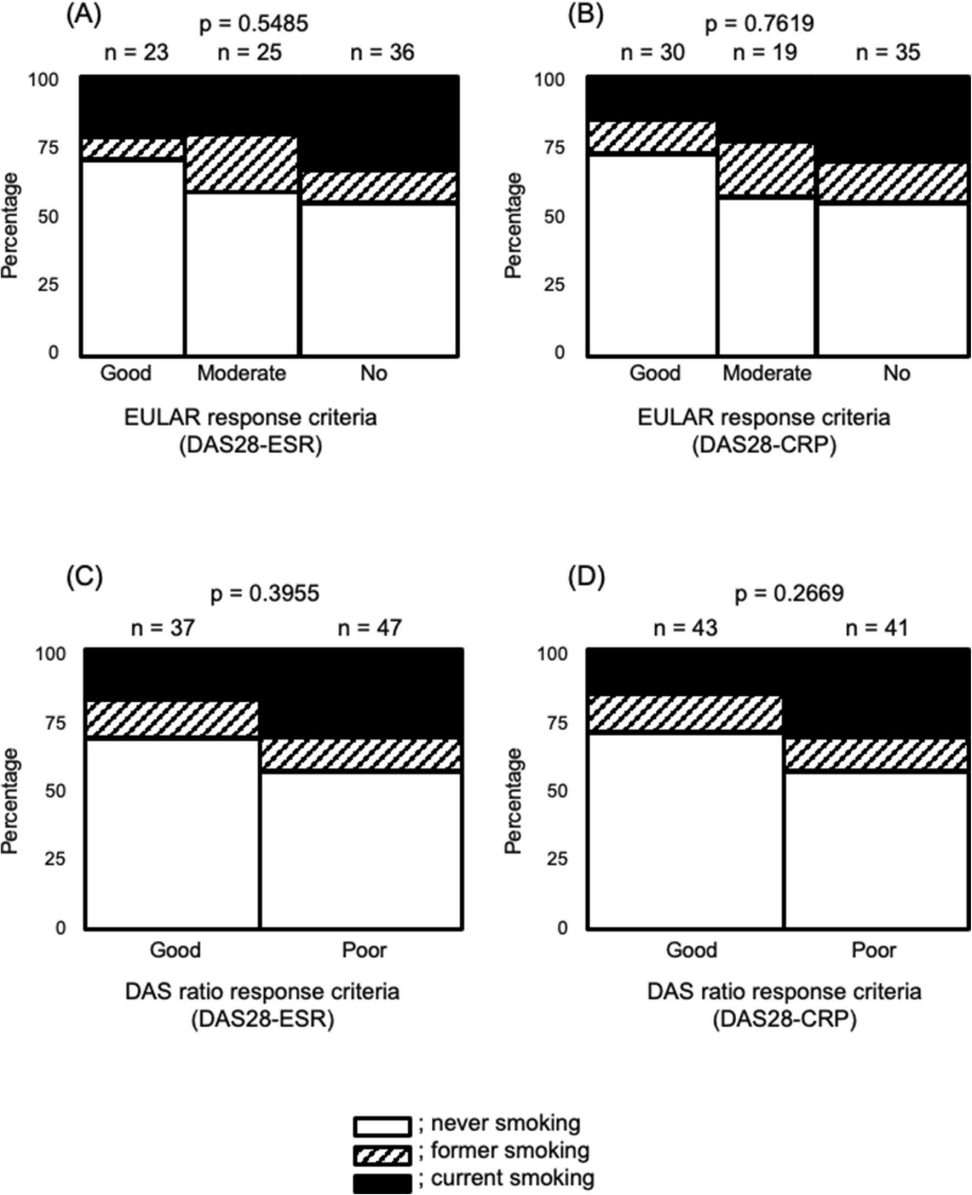
Relationship between therapeutic response and smoking. Shown in the Marimekko Chart (**A**,**B**) EULAR response criteria; (**C**,**D**) DAS ratio response criteria; (**A**,**C**) DAS28-ESR; (**B**,**D**) DAS28-CRP. Open bar represents never smoking; shaded bar represents former smoking; closed bar represents current smoking. Good responders had little smoking experience; however, there was no significant difference between groups (P-value: Pearson’s chi-square test). *CRP* C-reactive protein, *DAS* disease activity score, *ESR* erythrocyte sedimentation rate, *EULAR* European Alliance of Associations for Rheumatology.

Patients’ age was negatively correlated with ACPA levels and positively correlated with DAS28 but not with periodontal bacterial infection (Supplemental Table S1). Smoking status was not correlated with either RA or periodontal disease parameters (Supplemental Table S2 and Supplemental Fig. S4). In addition, there was no difference in history of smoking between the groups divided by disease (PMR or RA), degree of ACPA titers, and disease activity (Tables 2, 3, 4). There were also no significant differences between smoking and therapeutic responses; however, patients who had never smoked tended to have a better response to RA treatment (Fig. 2).

**Table 2:** Serum IgG antibody titers of patients with PMR and RA.

		PMR (*n* = 38)	RA (*n* = 82)	P-value
**Smoking**	Current	3 (7.9%)	12 (14.6%)	0.4811^a^ (0.2670)^b^
	Former	7 (18.4%)	18 (22.0%)	
	Never	28 (73.7%)	52 (63.4%)	
*Aa* titer		0.98 ± 0.27 (0.93)	1.11 ± 0.19 (0.79)	0.6930
*Pg titer*		0.62 ± 0.60 (0.42)	1.63 ± 0.40 (0.38)	0.1635

Data are expressed as means ± SE (median).

*Aa Aggregatibacter actinomycetemcomitans*, *IgG* immunoglobulin G, *Pg Porphyromonas gingivalis*, *PMR* polymyalgia rheumatic, *RA* rheumatoid arthritis, *SE* standard error.

There were no significant differences in antibody titers between the PMR and RA groups.

Effect of smoking was tested using Fisher's exact test for "a" and Pearson’s chi-square test for "b".

^a^Comparison between each value (3 × 2 Fisher’s exact test).

^b^Comparison between "current + former" vs "never.” (2 × 2 Pearson’s chi-square test).

**Table 3:**
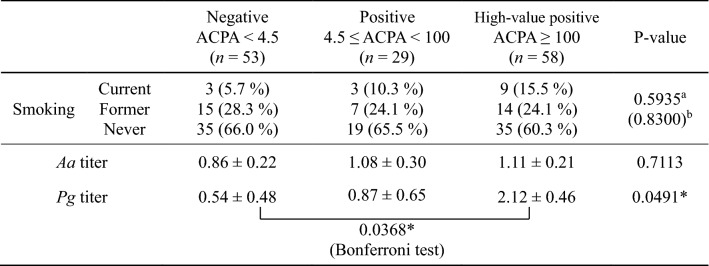
Serum IgG antibody titers based on the degree of ACPA titers.

Data are expressed as means ± SE.

*Aa Aggregatibacter actinomycetemcomitans*, *ACPA* anti-citrullinated peptide antibody, *IgG* immunoglobulin G, *Pg Porphyromonas gingivalis*, *SE* standard error.

The antibody titers against *Aa* did not differ significantly depending on the degree of ACPA titers, but the antibody titer against *Pg* showed a significantly high value as the degree of ACPA titers (P-value = 0.0491: one-way analysis of variance, *P-value < 0.05). There was also a significant difference in *Pg* when comparing the negative group and the high-value ACPA positive group (P = 0.0368: Bonferroni test, *P-value < 0.05).

Effect of smoking was tested using Fisher's exact test for "a" and Pearson’s chi-square test for "b".

^a^Comparison between each value (3 × 3 Fisher's exact test).

^b^Comparison between "current + former" vs "never.” (2 × 3 Pearson’s chi-square test).

**Table 4:** Serum IgG antibody titers by disease activity.

**(A) DAS28-ESR**					
		Low activity 2.6 ≤ DAS28 < 3.2 (*n* = 10)	Moderate activity 3.2 ≤ DAS28 < 5.1 (*n* = 45)	High activity DAS28 ≥ 5.1 (*n* = 31)	P-value
Smoking	Current	1 (10.0%)	5 (11.1%)	6 (19.4%)	0.3372^a^ (0.1339)^b^
	Former	3 (30.0%)	8 (17.8%)	10 (32.3%)	
	Never	6 (60.0%)	32 (71.1%)	15 (48.4%)	
*Aa* titer		0.58 ± 0.58	0.96 ± 0.27	1.15 ± 0.33	0.6892
*Pg* titer		− 0.23 ± 1.37	1.63 ± 0.64	1.63 ± 0.78	0.4422

**(B) DAS28-CRP**					
		Low activity 2.3 ≤ DAS28 < 2.7 (*n* = 6)	Moderate activity 2.7 ≤ DAS28 < 4.1 (*n* = 41)	High activity DAS28 ≥ 4.1 (*n* = 34)	P-value
Smoking	Current	0 (0.0%)	11 (26.8%)	10 (29.4%)	0.3316^a^ (0.2629)^b^
	Former	1 (16.7%)	4 (9.8%)	7 (20.6%)	
	Never	5 (83.3%)	26 (63.4%)	17 (50.00%)	
*Aa* titer		0.45 ± 0.78	0.84 ± 0.30	1.44 ± 0.33	0.2875
*Pg* titer		0.58 ± 1.83	1.66 ± 0.70	1.47 ± 0.77	0.8576

Data are expressed as means ± SE.

*Aa Aggregatibacter actinomycetemcomitans*, *CRP* C-reactive protein, *DAS28* disease activity score 28, *ESR* erythrocyte sedimentation rate, *IgG* immunoglobulin G, *Pg Porphyromonas gingivalis*, *SE* standard error.

There were no significant differences between the disease activities (P-value: one-way analysis of variance).

Effect of smoking was tested using Fisher's exact test for "a" and "b".

^a^Comparison between each value (3 × 3 Fisher's exact test).

^b^Comparison between "current + former" vs "never.” (2 × 3 Fisher's exact test).

### Serum IgG titers against periodontal pathogenic bacterial antigens

The averages and standard errors of serum IgG antibody titers in patients with PMR and RA are shown in Table 2. There were no significant differences in antibody titers between the PMR and RA groups. Serum IgG antibody titers against *Aa* were not significantly different according to the degree of ACPA titers; however, serum IgG titers against *Pg* were significantly higher in the group with a higher degree of ACPA titers (Table 3, p = 0.0491). There was also a significant difference in IgG titers against *Pg* between the ACPA-negative and high-value ACPA-positive groups (Table 3, p = 0.0368). Serum IgG antibody titers were not significantly different between DAS28-ESR and DAS28-CRP groups (Table 4). However, all 10 patients with low DAS28-ESR activity (Table 4A) and five of the six patients with low DAS28-CRP activity (Table 4B) had a low titer value (< 1) of serum IgG titers against *Pg*.

**Figure 3:**
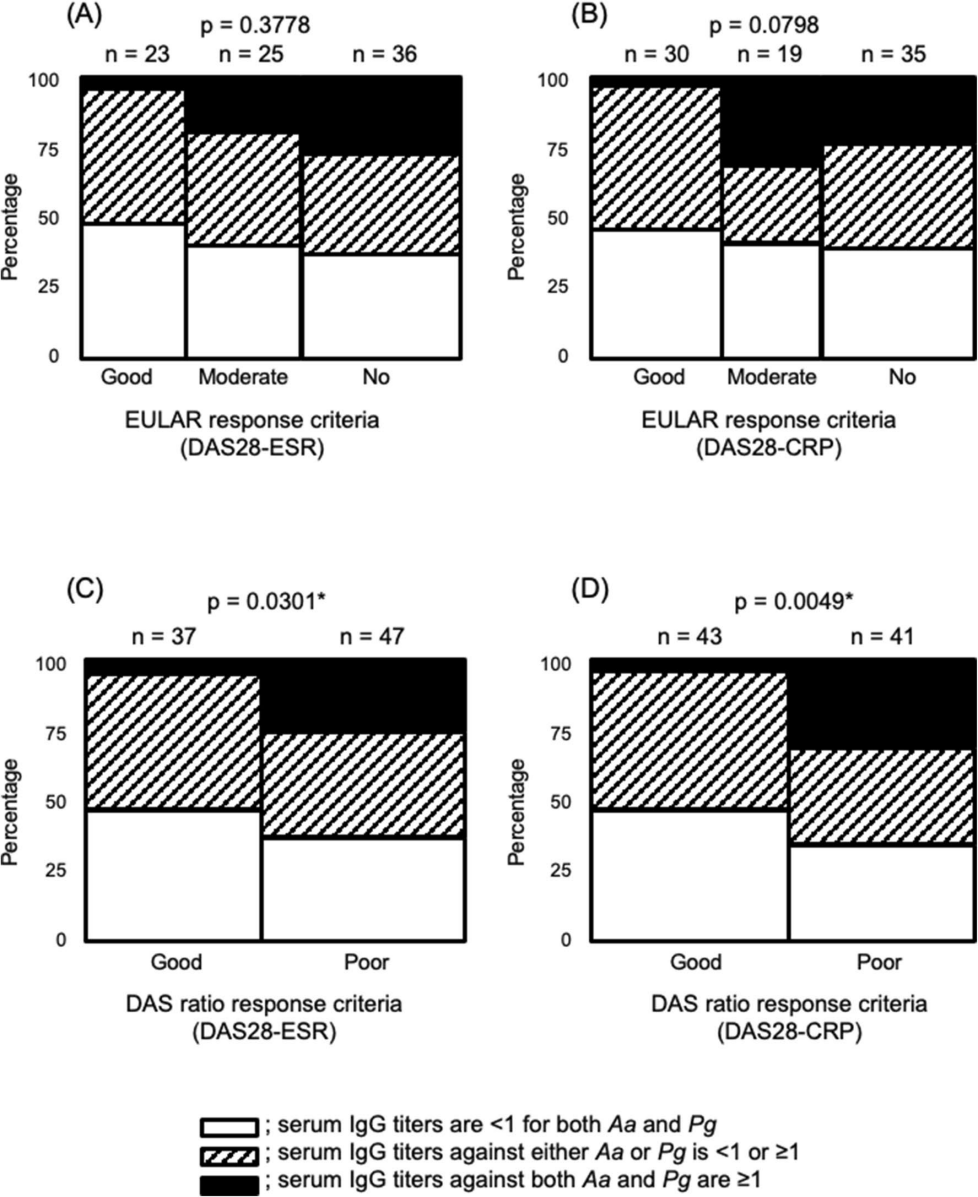
Relationship between therapeutic response and serum IgG antibody titers against *Aa* and *Pg*. Shown in the Marimekko Chart (**A**,**B**) EULAR response criteria; (**C**,**D**) DAS ratio response criteria; (**A**,**C**): DAS28-ESR; (**B**,**D**): DAS28-CRP. Open bar: serum IgG titers are < 1 for both *Aa* and *Pg*; shaded bar: serum IgG titers against either *Aa* or *Pg* is < 1 or ≥ 1; closed bar: serum IgG titers against both *Aa* and *Pg* are ≥ 1. Poor responders had high IgG titers against *Aa* and *Pg*. (P-value: Pearson’s chi-square test, * < 0.05). *Aa Aggregatibacter actinomycetemcomitans*, *CRP* C-reactive protein, *DAS* disease activity score, *ESR* erythrocyte sedimentation rate, *EULAR* European Alliance of Associations for Rheumatology, *IgG* immunoglobulin G, *Pg Porphyromonas gingivalis.*

The relationship between serum IgG titer against periodontal bacteria and therapeutic response is shown in Fig. 3. There were no significant differences between IgG antibody titers and therapeutic responses defined by the EULAR response criteria (Fig. 3AB). However, there were significant differences between IgG antibody titers and therapeutic responses defined by the DAS ratio at 0.75 calculated using DAS28-ESR (Fig. 3C, p = 0.0301) and DAS28-CRP (Fig. 3D, p = 0.0049). *Pg* titer was associated with therapeutic response (DAS28-CRP ratio of 0.75) with an odds ratio (95% CI) of 2.85 (1.12–7.27) and p-value of 0.0284. *Aa* titer was not significantly associated with an odds ratio (95% CI) of 1.78 (0. 740–4.27), with p-value of 0.198.

## Discussion

In the present study, serum antibody titers against periodontal bacteria related to citrullination were compared by the degree of ACPA titers among patients with RA and PMR. The antibody titer against *Pg* was significantly high in the ACPA-positive patients (Table 3). Although there was no statistically significant difference, the average antibody titer against *Pg* was higher in patients with RA than in those with PMR (Table 2). These results are consistent with the results of a previous report^15,37^, which compared patients with and without RA. Another study reported that ACPA-positive individuals had increased relative abundance of *Pg* but not *Aa*, suggesting that they may be targets for preventive intervention for RA^38^. Therefore, *Pg* infection may be considered to be associated with ACPA-positive RA. In addition, the severity of periodontitis has been reported to be significantly associated with RA activity^26,39^. Serum antibody titers against bacteria correlate with severity of periodontitis^40,41^. Because ACPA is related with *Pg* enzyme called peptidylarginine deiminase^42^, we focused on the infection severity of *Pg* by using IgG antibody titer. Thus, we hypothesized that antibody titers and RA activity might also be related. However, in our results it was not significantly associated with RA activity, but the IgG antibody titers against periodontal bacteria and the DAS28 score were roughly parallel and lower in the low activity group. Smoking is a major environmental risk factor for RA and has been reported in several meta-analyses^43,44^, however, no clear correlation between smoking and clinical parameters was found in our study (Supplemental Table S2 and Supplemental Fig. S4).

It is well known that the carrier rate of *Aa* is high in young individuals and decreases with age and that of *Pg* changes conversely^45^. A previous study reported that in the older group of individuals, antibody titer against *Pg* was elevated, while that against *Aa* was unchanged^46^. Our study found no correlation between age and antibody titers against *Aa* and *Pg* (Supplemental Table S1). Almost 80% of the population over 30 years of age experiences periodontal disease^47^. Our study population must have been old enough to present with a decreased infection of *Aa* and an increased infection of *Pg*. However, contrary to the previous reports, we found that most patients with RA and PMR had relatively low titers of anti-*Pg* antibodies and relatively high titers of anti-*Aa* antibodies (Tables 2, 3, 4)^35,48,49^. For example, it was reported that the median *Pg* titer of patients with chronic periodontitis was 1.28 for stable and 1.63 for progressive disease, whereas the median *Aa* titer was -0.26 for stable and -0.30 for progressive disease^49^. However, in the present study, the median *Pg* and *Aa* titers in patients with PMR were 0.42 and 0.93, respectively, and those in patients with RA were 0.38 and 0.79, respectively (Table 2). This may suggest that the immune response of patients with RA and PMR behaves in a similar manner and is somewhat different from the response of patients with periodontitis but without RA.

In addition, smokers have been reported to have decreased serum IgG antibody titers against periodontal bacteria^50^, suggesting a decrease in antibody-producing ability. However, no correlation between smoking and antibody titers was observed in our study (Supplemental Table S2).

When the relationship between the antibody titers against periodontal bacteria and the therapeutic response at 3 months after RA treatment was investigated, logistic analysis was conducted using *Aa* and *Pg* antibody titers, with therapeutic response as the objective variable. The results showed that a high antibody titer of *Pg* exceeding two SDs of the average titer in healthy individuals, was significantly poor response with an odds ratio of 2.85. On the other hand, there was no significant difference in *Aa* antibody titer. Furthermore, the RA patient group with poor therapeutic response had a high antibody titer against both *Pg* and *Aa* exceeding two SDs of the average titer in healthy individuals, whereas the patient group with good therapeutic response had a low antibody titer (Fig. 3). Periodontitis is a complex infection caused by multiple species of bacteria, and since the coexistence of *Pg* and *Aa* has been elucidated^51^, it may be worthwhile to analyze these two species together. This is a novel finding that infection with periodontal pathogenic bacteria seems to interfere with the therapeutic response of RA. Although all RA treatments for the patients in this study were conventional synthesis disease modifying anti rheumatic drug (csDMARDs) such as methotrexate or tacrolimus with or without prednisolone, it is essentially necessary to confirm that there is no significant difference in RA treatment regimen between the comparators. There may also be the influence of other factors such as differences in genetic background like SE and individual differences in humoral immune response. In contrast, we did not find a clear difference in the therapeutic response due to smoking in this study (Fig. 2). It has been reported that there is no difference in the mean DAS28 score at 48 and 102 weeks based on smoking status (p = 0.881)^52^, while non-smokers have a higher EULAR response rate than smokers or those with a history of smoking^53^.

This study had three major limitations. First, serum IgG antibody titers were measured only in the sera obtained before RA treatment. Second, the oral conditions of the target patients were not clear. Therefore, it is necessary that serum IgG antibody titers after RA treatment and fluctuations in APCA levels and disease activity after periodontal treatment are clarified in future studies. For example, Zhao et al.^54^ reported that patients with RA and periodontitis had significantly higher levels of CRP, ACPA, ESR, and DAS28 than those with RA without periodontitis. They suggested that nonsurgical periodontal treatment is effective in improving the clinical outcome of RA, and the routine use of this therapy is strongly recommended for patients with RA and periodontitis. Furthermore, nonsurgical periodontal therapy may aid in the control of RA-related autoimmune markers, such as serum ACPA in patients with chronic periodontitis^55^. However, controversial results have been reported in systematic reviews and open-label randomized controlled trials^56–58^. Thus, in third, randomized controlled trials and large-scale observational studies investigating the effect of periodontal bacterial infection on RA treatment response are needed in future. Although there are these limitations, it is our interests whether the therapeutic response can be predicted by the evaluation of infection of periodontal pathogenic bacteria. This is one of the benefits for the rheumatologists and physicians to screen the infection of periodontal pathogenic bacteria using blood test without any further dental examinations. This may allow physicians to predict the treatment resistance of RA related to this infection and consult periodontologists for precise examination of periodontitis.

Although we could not find any significant correlation between smoking and clinical features of RA, we found that *Pg* infection in periodontitis may be involved with the elevated levels of ACPA, and *Pg* and *Aa* infections may influence the therapeutic response of RA.

## Supplementary Information

Supplementary Figure 1.

Supplementary Figure 2.

Supplementary Figure 3.

Supplementary Figure 4.

Supplementary Table 1.

Supplementary Table 2.

Supplementary Legends.

## Data Availability

The datasets used and/or analyzed during the current study are available from the corresponding author, who has the ORCID identifier 0000-0002-4712-6829, on reasonable request.

## Abbreviations

*Aa*: *Aggregatibacter actinomycetemcomitans*
ACPA: Anti-citrullinated peptide antibody
ACR: American College of Rheumatology
CRP: C-reactive protein
DAS28: Disease activity score 28
ELISA: Enzyme-linked immunosorbent assay
ESR: Erythrocyte sedimentation rate
EULAR: European Alliance of Associations for Rheumatology
IgG: Immunoglobulin G
*Pg*: *Porphyromonas gingivalis*
PMR: Polymyalgia rheumatica
RA: Rheumatoid arthritis
RF: Rheumatoid factor
SD: Standard deviation
SE: Standard error
