# Supplementary figures and images for: Treatment resistance of rheumatoid arthritis relates to infection of periodontal pathogenic bacteria: a case–control cross-sectional study
Source: Sci Rep. 2022 Jul 19;12:12353. doi: 10.1038/s41598-022-16279-z (PMC9296452; doi:10.1038/s41598-022-16279-z)

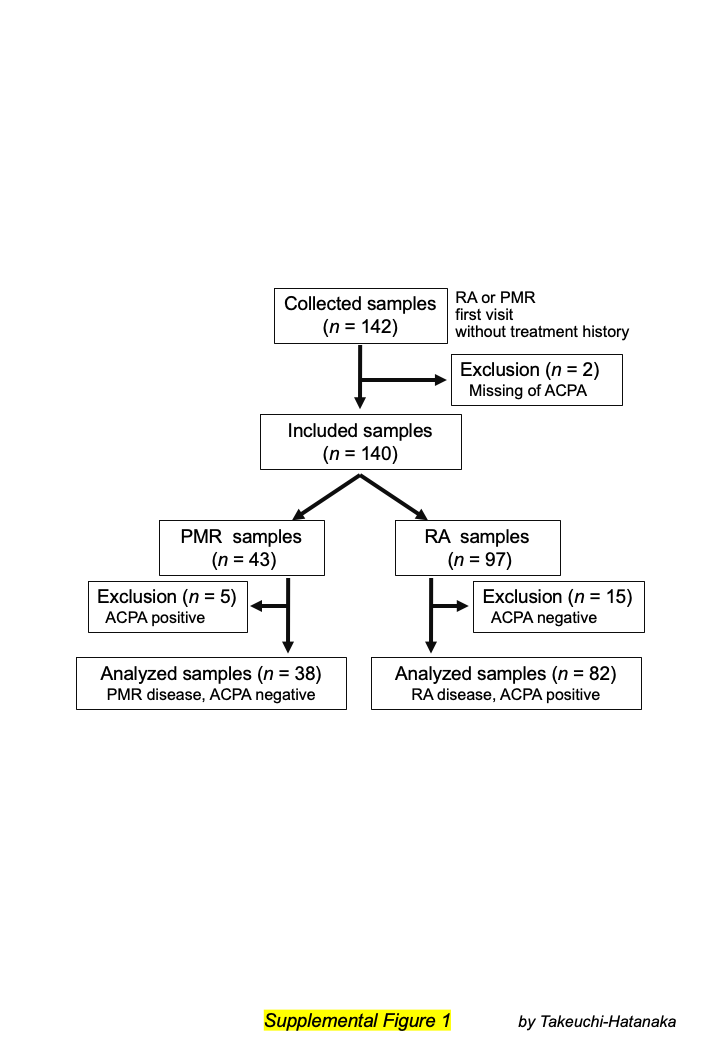

Supplement: Supplementary file 1 — Supplementary Figure 1. [file 41598_2022_16279_MOESM1_ESM.tiff]

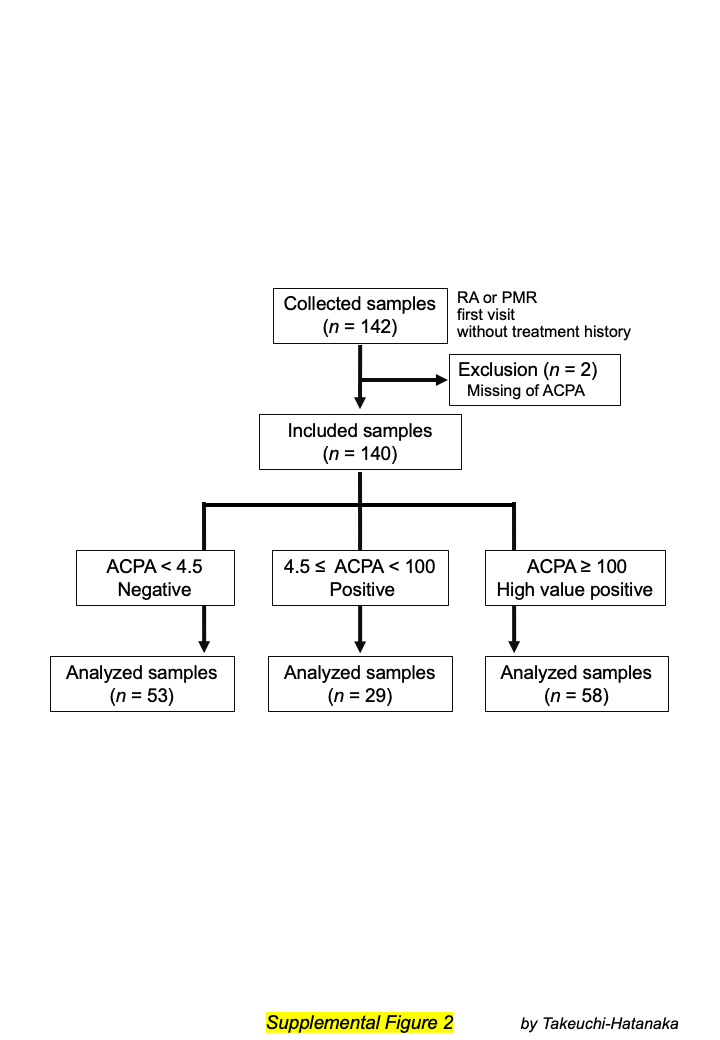

Supplement: Supplementary file 2 — Supplementary Figure 2. [file 41598_2022_16279_MOESM2_ESM.tiff]

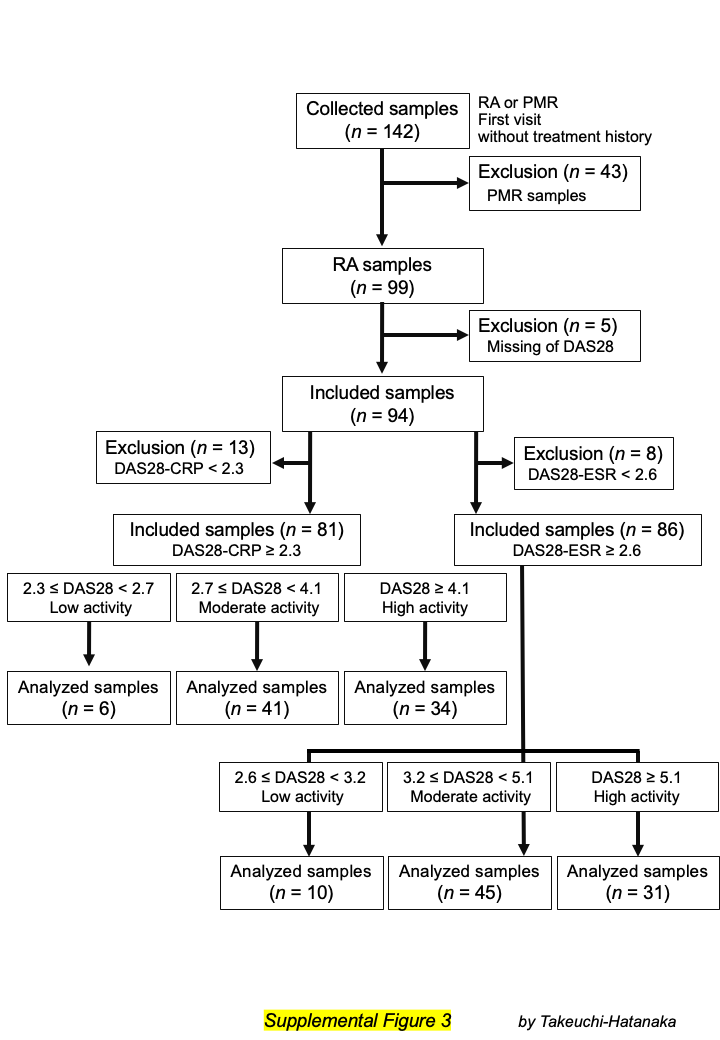

Supplement: Supplementary file 3 — Supplementary Figure 3. [file 41598_2022_16279_MOESM3_ESM.tiff]

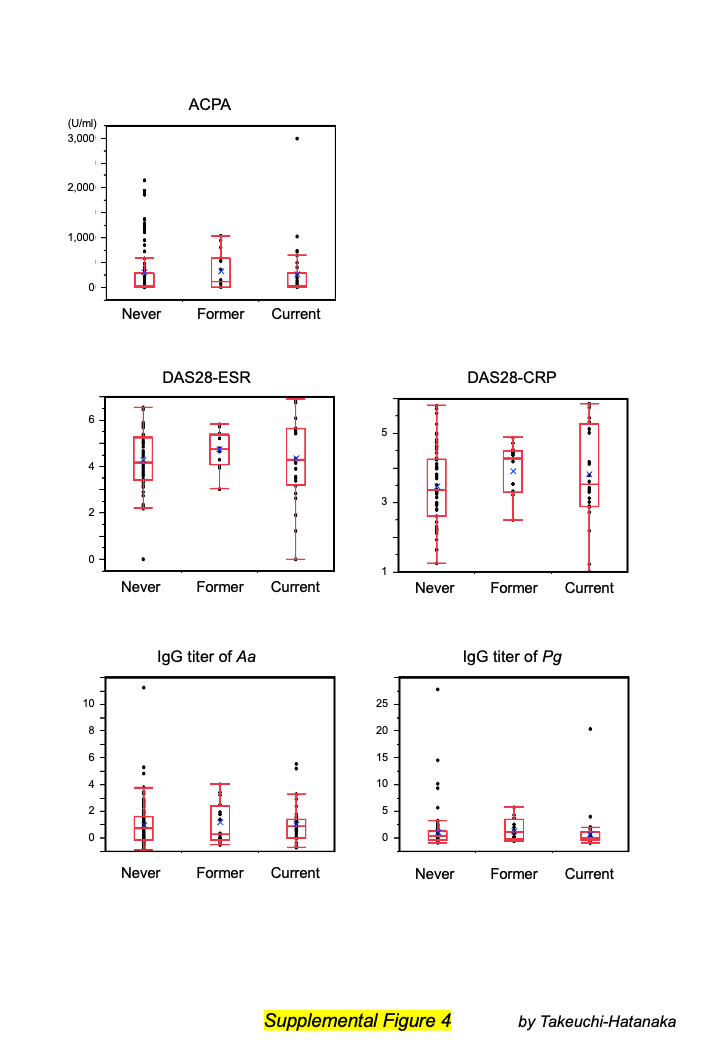

Supplement: Supplementary file 4 — Supplementary Figure 4. [file 41598_2022_16279_MOESM4_ESM.tiff]
