# Supplementary material for: Treatment resistance of rheumatoid arthritis relates to infection of periodontal pathogenic bacteria: a case–control cross-sectional study
Source: Sci Rep. 2022 Jul 19;12:12353. doi: 10.1038/s41598-022-16279-z (PMC9296452; doi:10.1038/s41598-022-16279-z)
Supplement: Supplementary file 5 — Supplementary Table 1. [file 41598_2022_16279_MOESM5_ESM.docx]

***Supplemental Table 1.* Relationship between patient age and clinical parameters**

|  | *r* | P-value |
| --- | --- | --- |
| ACPA | -0.3315 | 0.0001* |
| DAS28-ESR | 0.2394 | 0.0201* |
| DAS28-CRP | 0.2147 | 0.0377* |
| *Aa* titer | -0.0770 | 0.3626 |
| *Pg* titer | -0.0292 | 0.7305 |

The parameters for rheumatoid arthritis were correlated with age while the those for periodontal disease were not (P-value: Spearman’s correlation test, *P-value <0.05).

*Aa*: *Aggregatibacter actinomycetemcomitans*; ACPA: anti-citrullinated peptide antibody; CRP: C-reactive protein; DAS28: Disease activity score 28; ESR: erythrocyte sedimentation rate; *Pg*: *Porphyromonas gingivalis*
