# Supplementary material for: Treatment resistance of rheumatoid arthritis relates to infection of periodontal pathogenic bacteria: a case–control cross-sectional study
Source: Sci Rep. 2022 Jul 19;12:12353. doi: 10.1038/s41598-022-16279-z (PMC9296452; doi:10.1038/s41598-022-16279-z)
Supplement: Supplementary file 6 — Supplementary Table 2. [file 41598_2022_16279_MOESM6_ESM.docx]

***Supplemental Table 2*. Relationship between smoking and clinical parameters**

|  | *r* | P-value |
| --- | --- | --- |
| ACPA | 0.0282 | 0.7407 |
| DAS28-ESR | 0.1012 | 0.3316 |
| DAS28-CRP | 0.1517 | 0.1445 |
| *Aa* titer | 0.0259 | 0.7597 |
| *Pg* titer | -0.0343 | 0.6855 |

None of the parameters were correlated with smoking (P-value: Spearman’s correlation test).

*Aa*: *Aggregatibacter actinomycetemcomitans*; ACPA: anti-citrullinated peptide antibody; CRP: C-reactive protein; rate; DAS28: Disease activity score 28; ESR: erythrocyte sedimentation; *Pg*: *Porphyromonas gingivalis*
