# Supplementary material for: Treatment resistance of rheumatoid arthritis relates to infection of periodontal pathogenic bacteria: a case–control cross-sectional study
Source: Sci Rep. 2022 Jul 19;12:12353. doi: 10.1038/s41598-022-16279-z (PMC9296452; doi:10.1038/s41598-022-16279-z)
Supplement: Supplementary file 7 — Supplementary Legends. [file 41598_2022_16279_MOESM7_ESM.docx]

**Supplemental Figure 1. Flow chart of total analyzed samples based on disease**

Serum IgG antibody titers in patients with PMR and RA were analyzed. Thirty-eight samples from patients with PMR were ACPA negative and 82 samples from patients with RA were ACPA positive.

ACPA: anti-citrullinated peptide antibody; IgG: immunoglobulin G; PMR: polymyalgia rheumatica; RA: rheumatoid arthritis

**Supplemental Figure 2. Flow chart of total analyzed samples based on degree of ACPA titers**

The serum IgG antibody titers were classified into three groups according to the degree of ACPA titers and analyzed. The ACPA level was <4.5 U/mL in 53 samples, ≥4.5 U/mL or <100 U/mL in 29 samples, and ≥100 U/mL in 58 samples.

ACPA: anti-citrullinated peptide antibody; IgG: immunoglobulin G

**Supplemental Figure 3. Flow chart of analyzed RA samples based on disease activity**

Serum IgG antibody titers were classified into three groups based on the DAS28 score and analyzed. DAS28-CRP was ≥2.3 and <2.7 in 6 samples, ≥2.7 and <4.1 in 41 samples, and ≥4.1 in 34 samples. DAS28-ESR was ≥2.6 and <3.2 in 10 samples, ≥3.2 and <5.1 in 45 samples, and ≥5.1 in 31 samples.

CRP: C-reactive protein; DAS28: Disease activity score 28; ESR: erythrocyte sedimentation rate; IgG: immunoglobulin G; RA: rheumatoid arthritis
